# Enantioselective Determination of Polycyclic Musks in River and Wastewater by GC/MS/MS

**DOI:** 10.3390/ijerph13030349

**Published:** 2016-03-22

**Authors:** Injung Lee, Anantha-Iyengar Gopalan, Kwang-Pill Lee

**Affiliations:** 1 Nakdong River Environment Research Center, National Institute of Environment Research, Gyoungbuk 717-873, Korea; ijlee5344@korea.kr; 2 Department of Chemistry Graduate School, Kyungpook National University, Daegu 702-701, Korea; algopal99@gmail.com; 3 Research Institute of Advanced Energy Technology, Kyungpook National University, Daegu 702-701, Korea

**Keywords:** chiral pollutants, polycyclic musks, river and wastewaters, Korea, enantioselective transformation

## Abstract

The separation of chiral compounds is an interesting and challenging topic in analytical chemistry, especially in environmental fields. Enantioselective degradation or bioaccumulation has been observed for several chiral pollutants. Polycyclic musks are chiral and are widely used as fragrances in a variety of personal care products such as soaps, shampoos, cosmetics and perfumes. In this study, the gas chromatographic separation of chiral polycyclic musks, 1,3,4,6,7,8-hexahydro-4,6,6,7,8,8-hexamethylcyclo-penta-γ-2-benzopyrane (HHCB), 7-acetyl-1,1,3,4,4,6-hexamethyl-1,2,3,4-tetra-hydronaphthalene (AHTN), 6-acetyl-1,1,2,3,3,5-hexamethylindane (AHDI), 5-acetyl-1,1,2,6-tetramethyl-3-*iso*-propylindane (ATII), and 6,7-dihydro-1,1,2,3,3-pentamethyl-4(5H)-indanone (DPMI) was achieved on modified cyclodextrin stationary phase (heptakis (2,3-di-*O*-methyl-6-*O-tert*-butyl-dimethylsilyl-β-CD in DV-1701)). Separation techniques are coupled to tandem mass spectrometry (MS-MS), as it provides the sensitivity and selectivity needed. River and wastewaters (influents and effluents of wastewater treatment plants (WWTPs)) in the Nakdong River were investigated with regard to the concentrations and the enantiomeric ratios of polycyclic musks. HHCB was most frequently detected in river and wastewaters, and an enantiomeric enrichment was observed in the effluents of one of the investigated wastewater treatment plants (WWTPs). We reported the contamination of river and wastewaters in Korea by chiral polycyclic musks. The results of this investigation suggest that enantioselective transformation may occur during wastewater treatment.

## 1. Introduction

Chiral pollutants constitute a growing area of research in the field of environmental chemistry. Many chemicals of environmental interest and concern are chiral. Specifically, about 25% of all agrochemicals are chiral, as are many other common chemicals [1,2,3,4,5,6]. Enantiomers have identical physical and chemical properties. Thus, abiotic environmental processes (e.g., air-water exchange, sorption, abiotic transformation) are generally identical for stereoisomers. However, biochemical processes (e.g., biodegradation) and toxicological effects can be enantioselective because individual stereoisomers can interact differentially with other chiral molecules, such as enzymes and biological receptors. Thus, stereoisomers can have different biological and toxicological effects. Enantioselective investigations of chiral pollutants in environmental samples may give additional information on the accumulation of anthropogenic contaminants in the food web or possible degradation pathways and may allow a distinction to be made between enzymatic biotic and non-enzymatic abiotic processes [1,2,3,4,5,6]. 

Synthetic musks, as a new emerging pollutant, are widely used as fragrances in a variety of personal care products such as soaps, shampoos, cosmetics, and perfumes. The main classes of synthetic musks are polycyclic musks, which include 1,3,4,6,7,8-hexahydro-4,6,6,7,8,8-hexamethylcyclo-penta-γ-2-benzopyrane (HHCB), 7-acetyl-1,1,3,4,4,6-hexamethyl-1,2,3,4-tetra-hydronaphthalene (AHTN), 4-acetyl-1,1-dimethyl-6-*tert*-butylindane (ADBI), 6-acetyl-1,1,2,3,3,5-hexamethylindane (AHDI), 6,7-dihydro-1,1,2,3,3-pentamethyl-4(5H)-indanone (DPMI), and 5-acetyl-1,1,2,6-tetramethyl-3-*iso*-propylindane (ATII). They have been detected in municipal sewage effluent and various environmental samples such as surface water, marine, soil, sediment and aquatic biota in many countries. The occurrence of synthetic musks in environmental samples could negatively affect the health of the ecosystem and humans, owing to persistent, chronic exposure of aquatic organisms [7,8,9,10,11].

**Figure 1:**
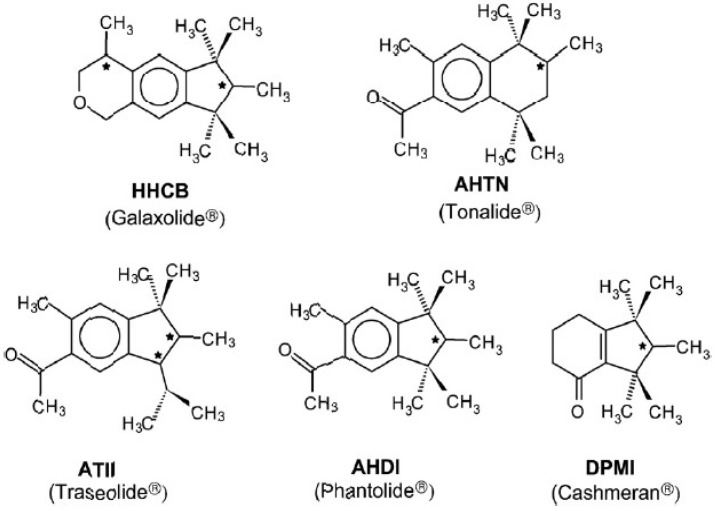
Chemical structures of the chiral polycyclic musks.

Polycyclic musks contain one (AHTN, AHDI, DPMI) or two (HHCB, ATII) chiral atoms. The chemical structures of these compounds are given in Figure 1. ADBI is not chiral. They largely occur as racemic mixtures in commercial products. The current industrial synthesis is racemic, though normally only one enantiomer, e.g., *S*-AHTN but not *R*-AHTN, is effective. Some compounds, such as HHCB, contain two chiral centers, and thus four stereoisomers are possible. However, only the two 4*S* stereoisomers are active in the human nose and are powerful, musky components [12,13,14,15,16,17,18]. These stereoisomers leave a different sensory impression. These enantioselectivity issues might become a topic in chemical regulation such as REACH (registration, evaluation, authorization and restriction of chemicals) as they already are in pesticides and pharmaceuticals [2,4,5,6].

The separation of chiral compounds is an interesting and challenging topic in analytical chemistry, especially in biomedical, pharmaceutical, and environmental fields. Owing to the similar physical and chemical properties of enantiomers, their resolution is very difficult. The techniques frequently used for chiral separations include gas chromatography (GC), liquid chromatography (LC), capillary electrophoresis (CE) and capillary electrochromatography (CEC) using a chiral selector [1,2,5,6,19,20]. GC has been widely used for the determination of some chiral environmental pollutants (e.g., polycyclic musks, 1,1,1-trichloro-2,2-bis-(4-chlorophenyl)ethane (DDT), polychlorinated biphenyls (PCBs), and chlordans) using the chiral stationary phase [1,2,5,6]. Cyclodextrins (CDs), especially β-CD, are the most popular chiral stationary phase in enantiomeric separation via inclusion complexation. Besides the native CD, functionalized CDs such as hydroxypropyl-β-CD, permethyl-β-CD, *tert*-butyldimethylsilylated β-CD, and (2,3-di-*O*-methyl-6-*O*-*tert*-butyldimethylsilyl-)-β-CD have also been used as chiral stationary phases to expand the range of compounds that can be resolved enantioselectively [1,2,5,6,20,21]. Separation techniques in environmental analysis are typically coupled to mass spectrometry (MS) and tandem mass spectrometry (MS-MS), as these provide the required sensitivity and selectivity [1,2,5,6]. 

In this study, the gas chromatographic separation of several chiral polycyclic musks was achieved in cyclodextrin phases. Separation techniques are coupled to MS-MS, as it provides the sensitivity and selectivity needed. The concentrations and enantiomeric ratios (ERs) of HHCB, AHTN, ATII, AHDI, and DPMI were determined in the river and wastewater samples from the Nakdong River, Korea. There are very few reports about the occurrence and environmental behavior of chiral pollutants in Korea.

## 2. Materials and Methods

### 2.1. Chemicals and Apparatus

*n*-Hexane and dichloromethane were of analytical grade and were purchased from Merck (Darmstadt, Germany). Racemic HHCB, AHTN, ADBI, ATII and DPMI for the preparation of analytical standards were obtained from Dr. Ehrenstorfer GmbH (Augsburg, Germany). Anhydrous sodium sulfate was obtained from Merck and baked at 450 °C for 4 h prior to use. Water was purified by a Barnstead Nanopure Diamond UV water purification system. Evaporation of the solvent was accomplished with a TURBOVAP II concentrator (Zymark, MA, USA). All glassware was rinsed with acetone and n-hexane and baked at 450 °C for 2 h prior to use.

### 2.2. Characterization of Monitoring Sites and Sampling

**Figure 2:**
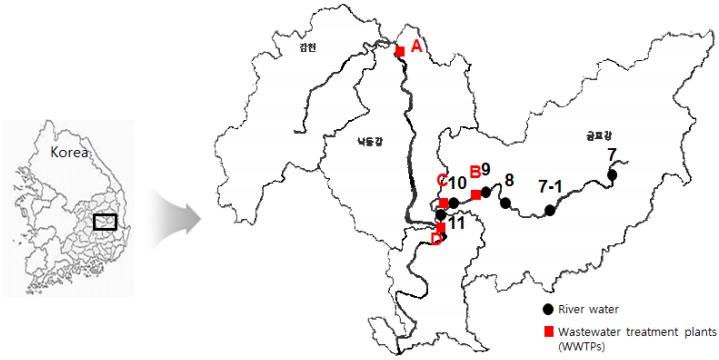
Description of river water and wastewater treatment plants sampling sites.

River water samples were collected at six sites of the monitoring network in the Nakdong River, a large river in southeast Korea. The sampling locations are described in Figure 2. Wastewater samples were collected from the influent and effluent of four wastewater treatment plants (WWTPs) in the Nakdong River basin (Plants A–D) (Figure 2). Table 1 presents the WWTPs information, including the wastewater capacity and the treatment processes. All WWTPs receive domestic and industrial discharge and were equipped with secondary treatment by activated sludge process. Samples were collected in July and September of 2013 using the grab sampling method. All samples were collected in 1 L pre-cleaned amber glass bottles and stored in the dark at 4 °C until they were extracted. All samples were analyzed within two weeks after collection. Field blanks and duplicates were taken at the time of sampling to check for contamination.

### 2.3. Sample Preparation

Polycyclic musks in river and wastewater samples were analyzed according to the method described in our previous paper [22,23]. Unfiltered water samples (100 mL for wastewater influent samples; 500 mL for other samples) were extracted with 50 mL of dichloromethane/*n*-hexane (1:1, v/v) in a glass separatory funnel. The mixture was agitated vigorously for 2 min and was then allowed to settle until the phases were fully separated. Anhydrous sodium sulfate was added to the extract to remove any remaining water. All extractions were repeated three times and the extracts were combined and concentrated under nitrogen. The final volume of the extract was adjusted to 0.5 mL with dichloromethane.

### 2.4. Quantitative GC-MS-MS Analysis

Five polycyclic musks were quantified by a gas chromatograph interfaced with a triple-quadrupole mass spectrometric detector (GC-MS-MS, Varian 450-GC and 300 TQ-MSD; Varian AG, Zug, Switzerland) under the following conditions: column, VM5-MS (30 m × 0.25 mm × 0.25 μm); oven temperature program, 100 °C for 2 min, 10 °C/min to 200 °C, hold for 10 min and then 10 °C/min to 250 °C hold for 10 min; injection, 2 μL *via* splitless mode; carrier gas, helium 1.0 mL/min at constant flow; injector temperature, 250 °C; ion source temperature, 200 °C; transfer line temperature, 230 °C; ionization, electron impact mode with an potential of 70 eV. For quantification of the compounds, data were acquired in multiple reaction monitoring (MRM) mode using the characteristic ions given in Table 2.

The method was validated for quantitative measurements, in terms of limit of detection (LOD), limit of quantitation (LOQ), linearity of calibration curve, accuracy, and precision. Calibration curves for the analytes were obtained by extraction of the standard in the range between 0.1 and 2 μg/L. Good linearity was obtained for all target compounds. The LOD and LOQ were calculated from the standard deviation of the seven replicated analyses of spiked solution with a low concentration (0.1 μg/L). The LOD and LOQ ranged from 0.018 to 0.034, and from 0.058 to 0.107 μg/L, respectively. The accuracy and precision were determined by spiking solutions of five replicates (1 μg/L). The accuracy was acceptable and the relative standard deviations (RSDs) revealed an acceptable repeatability for all analytes. The method quality data is presented in Table 3.

### 2.5. Enantioselective GC-MS-MS Analysis

Enantioselective separation was performed with heptakis(2,3-di-*O*-methyl-6-*O-tert*-butyl-dimethylsilyl-β-CD) in DV-1701 column (Cyclosil-B, 25 m × 0.25 mm × 0.25 μm) as chiral stationary phase using the same GC-MS-MS equipment as described above and the same samples were used for quantification without a further purification. Separation of the enantiomers was achieved using the following temperature program: 100 °C, 2 min hold, 2 °C/min to 200 °C, 10 min hold. Helium was used as a carrier gas in the constant flow mode (1.0 mL/min). Standards and extracts were injected (1 μL) in splitless mode. Data was acquired in the MRM mode using the same ions as described above for quantification. MS temperature parameters are as follows: ion source, 200 °C; transfer line, 230 °C. 

## 3. Results and Discussion

### 3.1. Enantioselective Analysis of Polycyclic Musks

**Figure 3:**
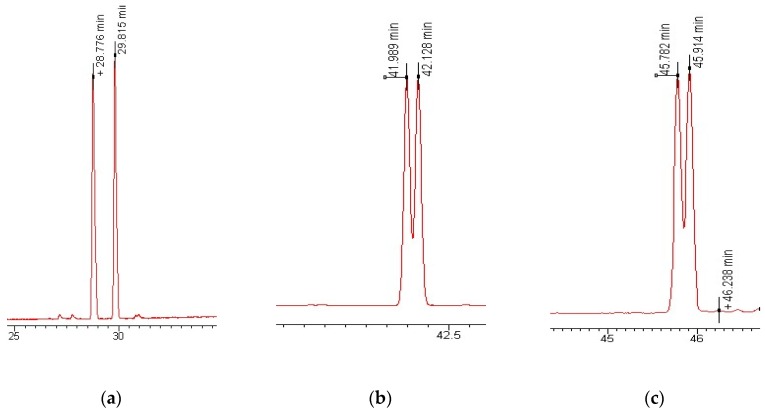

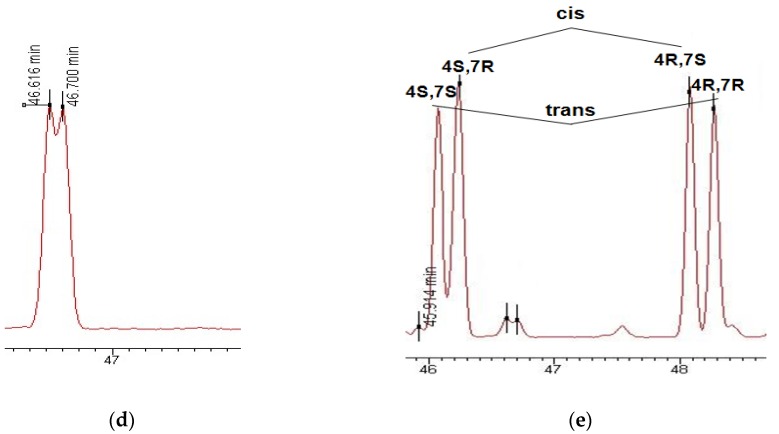
MRM chromatograms of chiral poycyclic musks using heptakis 2,3-di-*O*-methyl-6-*O-tert*-butyl-dimethylsilyl-β-CD in DV-1701 column. (**a**) Ion 191 for DPMI; (**b**) Ion 229 for AHDI; (**c**) Ion 215 for ATII; (**d**) Ion 187 for AHTN; (**e**) Ion 213 for HHCB.

In order to determine the ERs in river and wastewater samples, an enantioselective GC was used which facilitated the separation of enantiomeric or diastereomeric polycyclic musks with sufficient resolution. The gas chromatographic separation of chiral polycyclic musks, HHCB, AHTN, AHDI, ATII, and DPMI, was achieved on modified CD stationary phase (heptakis (2,3-di-*O*-methyl-6-*O-tert*-butyl-dimethylsilyl-β-CD in DV-1701)). The resolution factor was 1 for DPMI and HHCB, and 0.7–0.8 for AHDI and ATII, demonstrating that the corresponding peak pairs were well separated. The elution order of the HHCB stereoisomers was described previously [12,13,16,17,18,19]. HHCB was resolved into two widely spaced pairs of peaks, where the (*4S*, *7RS*)-diastereomers constituted the first pair and the (*4R*, *7RS*)-diastereomers composed the second pair. Thus, the pairs *4S*, *7S/4R*, *7R* and *4S*, *7R/4R*, *7S* were suggested to be enantiomers. MRM chromatograms for selected ions are shown in Figure 3. Optimization of the enantioselective GC separation was performed with an emphasis on temperature programming and initial temperature. The optimum resolution of the polycyclic musks was obtained by a slow temperature programming of 2 °C/min and the initial temperature was set to 100 °C to achieve adequate retention times. 

### 3.2. Enantiomeric Ratios of Polycyclic Musks in River and Wastewater

Frequently, the ER is taken as a parameter to describe enantioselective transformations. It is assumed that an ER close to 1 indicates a low transformation potential of a system whereas ER values different from 1 are a result of enantioselective transformations [1]. Polycyclic musks in raw wastewater are mostly racemic, suggesting little stereoselective degradation prior to environmental release, whereas wastewater effluents are non-racemic according to previous studies [14,17,18]. It can therefore be concluded that enantioselective transformation might occur during wastewater treatment. Franke *et al.* described an enantioselective and species-dependent transformation of HHCB and AHTN in the aquatic environment [13,15,16]. 

The concentrations and ERs of HHCB, AHTN, ATII, AHDI, and DPMI in river and wastewater samples are summarized in Table 4. Five polycyclic musks were quantified by GC-MS-MS using a nonchiral VM5-MS column. The ER values of DPMI, ATII, AHDI, and AHTN could not be calculated owing to the low amounts in the samples. In the river water samples, the HHCB composition was almost identical and was nearly racemic, but did not match exactly. For HHCB, significant deviations of the ER value from racemic was observed in the effluent of WWTP C for the *trans*- and *cis*-isomers. However, other WWTPs did not seem to induce enantioselective biotransformations in the compounds during the water treatment. It can be inferred that biotransformations might contribute to their removal of polycyclic musks from wastewater, in addition to sorption on sludge [13,14,15,16,17,18]. However, the exact mechanisms responsible for the significant deviations of the ER values in some environmental samples remain to be elucidated in the future.

## 4. Conclusions

Many environmental pollutants are chiral, used as a single enantiomer or as mixtures of the two enantiomers. In spite of their similar physical and chemical properties, the different spatial configurations lead to different interactions with enzymes, receptors, or other chiral molecules, yielding diverse biological responses. Many environmental pollutants are released into the environment as racemates, but frequently undergo alterations in their enantiomeric composition as soon as they are subjected to certain biochemical processes. The enantioselective analysis of chiral environmental pollutants is important, since enantiomers of chiral compounds often exhibit different biological activities, and most biochemical processes in nature are stereospecific. The chiral separation of enantiomers is one of the most challenging tasks for any analytical technique. Polycyclic musks are chiral and they are widely used as fragrances in a variety of personal care products such as soaps, shampoos, cosmetics and perfumes. In this study, the gas chromatographic separation of chiral polycyclic musks, HHCB, AHTN, AHDI, ATII, and DPMI, was achieved on modified CD stationary phase (heptakis (2,3-di-*O*-methyl-6-*O-tert*-butyl-dimethylsilyl-β-CD in DV-1701)). Separation techniques coupled to MS-MS provide the required sensitivity and selectivity. The concentrations and ERs of HHCB, AHTN, ATII, AHDI, and DPMI were determined in river and wastewater samples from the Nakdong River, Korea. HHCB was most frequently detected in river and wastewaters, and an enantiomeric excess was observed in the effluents of WWTP C. The results of this investigation suggest that enantioselective transformation may occur during wastewater treatment as reported in the literature [13,14,15,16,17,18]. 

## Figures and Tables

**Table 1:** Description of the investigated WWTPs ^a^.

WWTPs ^a^	Location	Treatment (m^3^/day)	Area Served (km^2^)	Population Served	Treatment Processes
A	Chilgok	330,000	31.56	275,000	Activated sludge
B	Daegu	680,000	59.20	1,084,000	
C	Daegu	400,000	19.64	423,000	
D	Daegu	520,000	44.73	893,000	

^a^ WWTPs: Wastewater treatment plants.

**Table 2:** MRM ^a^ ions for the analysis of polycyclic musks.

Compounds	MRM ^a^ Ions		
	Parent	Daughter 1	Daughter 2
DPMI	206	191	163
AHDI	244	229	187
ATII	258	215	173
HHCB	258	243	213
AHTN	258	243	187

^a^ MRM: Multiple reaction monitoring.

**Table 3:** Method quality data for the quantification of polycyclic musks.

Compounds	Linearity ^a^ (r^2^)	LOD ^b^ (μg/L)	LOQ ^b^ (μg/L)	Accuracy ^c^ (%)	Precision ^c^ (RSD, %)
HHCB	0.984	0.018	0.058	78.2	14.1
AHTN	0.980	0.024	0.076	75.2	17.0
AHDI	0.988	0.022	0.069	79.0	15.9
DPMI	0.991	0.025	0.079	85.2	13.3
ATII	0.989	0.034	0.107	80.6	11.9

^a^ conc. range: 0.1–2 μg/L; ^b^ spiked conc.: 0.1 μg/L, *n* = 7, LOD (limit of detection) = SD × 3.14, LOQ (limit of quantitation) = SD × 10; ^c^ spiked conc.: 1 μg/L, *n* = 5.

**Table 4:** Concentrations and enantiomeric ratios (ERs) of chiral polycyclic musks in river and wastewater. (AHTN, ATII, AHDI, and DPMI were detected below the limit of quantitation (LOQ) in river and wastewater samples.)

Sample			HHCB		
			Concentration (μg/L)	ER	
				trans-HHCB	cis-HHCB
River water	7	1st	n.d ^a^	-	-
		2nd	n.d	-	-
	7–1	1st	n.d	-	-
		2nd	n.d	-	-
	8	1st	0.277	0.95	1.03
		2nd	n.d	-	-
	9	1st	n.d	-	-
		2nd	0.280	1.09	0.95
	10	1st	0.280	0.86	1.02
		2nd	0.342	0.97	1.10
	11	1st	n.d	-	-
		2nd	0.319	0.96	1.07
WWTPs	A	Influent	0.785	0.94	1.04
		Effluent	0.284	0.99	1.15
	B	Influent	0.998	1.01	1.14
		Effluent	0.351	1.04	1.18
	C	Influent1	1.150	0.91	0.98
		Influent2	2.016	0.98	1.02
		Effluent	0.289	0.74	0.69
	D	Influent	3.491	0.97	1.03
		Effluent	0.576	0.98	1.25

^a^ n.d.: not detected.
